# Supplementary material for: Does a rare mutation in PTPRA contribute to the development of Parkinson’s disease in an Australian multi-incident family?
Source: PLoS One. 2022 Jul 28;17(7):e0271499. doi: 10.1371/journal.pone.0271499 (PMC9333306; doi:10.1371/journal.pone.0271499)

Fig. S1

EGF treated cells do not have enhanced pSRC^{Y416} activity. Immunoblots of HEK293, HEK293-RPTPα^{WT} and HEK293-RPTPα^{R223W} cells probed with the indicated antibodies.

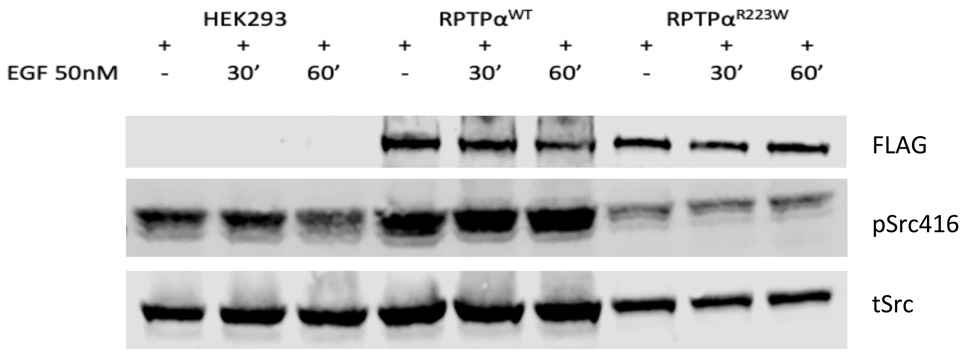

Supplement: S1 Fig — (PDF) [file pone.0271499.s001.pdf]
