# Supplementary material for: Does a rare mutation in PTPRA contribute to the development of Parkinson’s disease in an Australian multi-incident family?
Source: PLoS One. 2022 Jul 28;17(7):e0271499. doi: 10.1371/journal.pone.0271499 (PMC9333306; doi:10.1371/journal.pone.0271499)

Does a rare mutation in PTPRA contribute to the development of Parkinson’s disease in an Australian multi-incident family?

Hill MA^1^, Bentley SR^1^, Walker TL^2^, Mellick GD^1^, Wood SA^1^ and Sykes AM^1^

^1^ Griffith Institute for Drug Discovery, Griffith University, Nathan, Australia

^2^ Queensland Brain Institute, University of Queensland, Australia

**Supplementary Information: Original uncropped western blots**

Fig. 2A Anti-V5


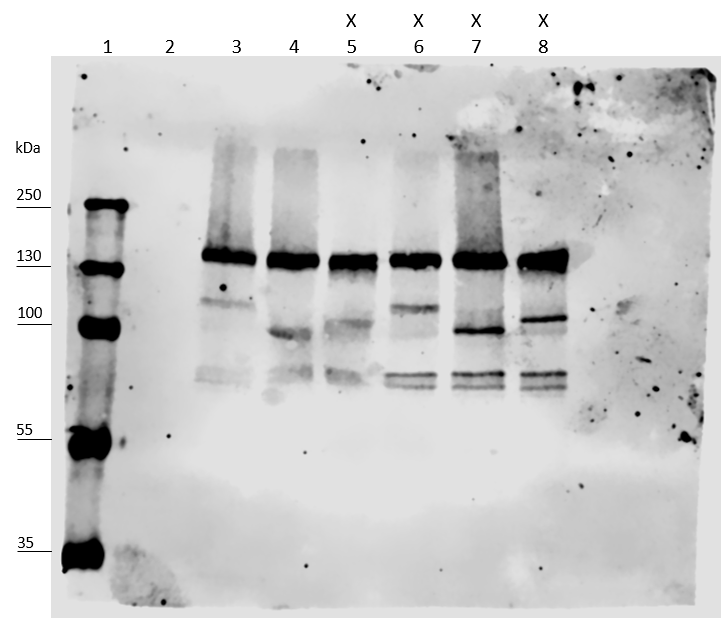


Fig. 2A Anti-α-tubulin.


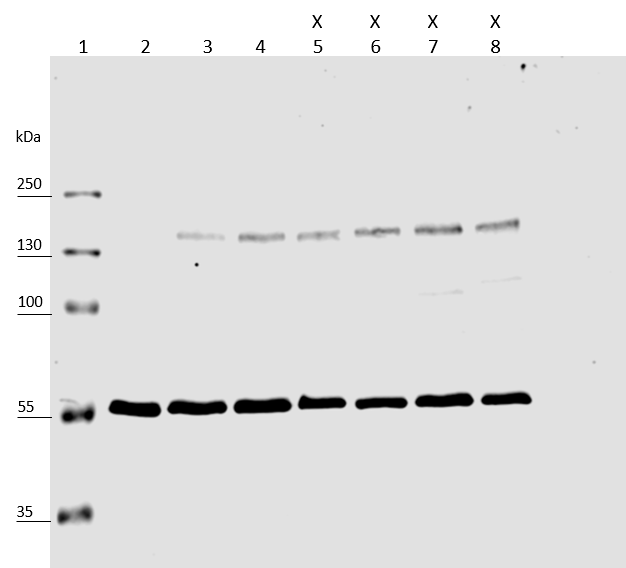


Fig. 2B Anti-V5 Fig. 2B Anti-α-tubulin


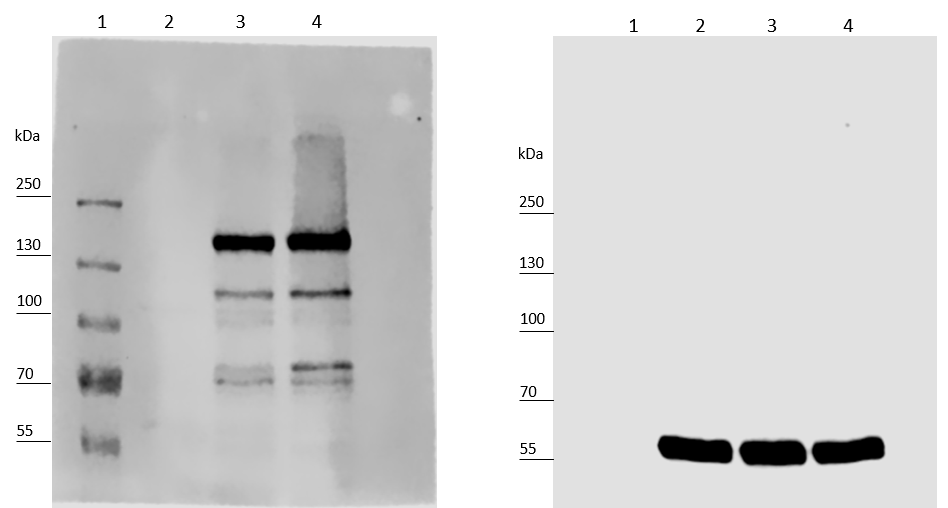


Fig. 3A Anti-V5


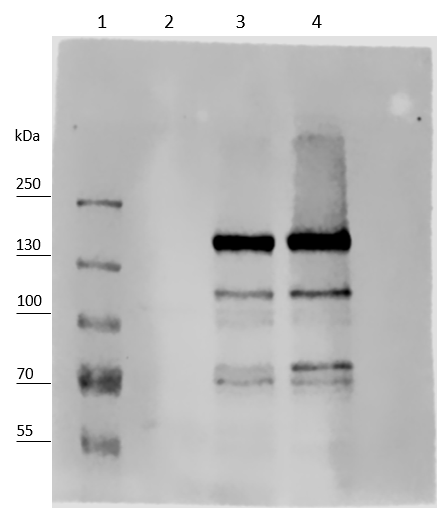


Fig. 3B Anti-V5


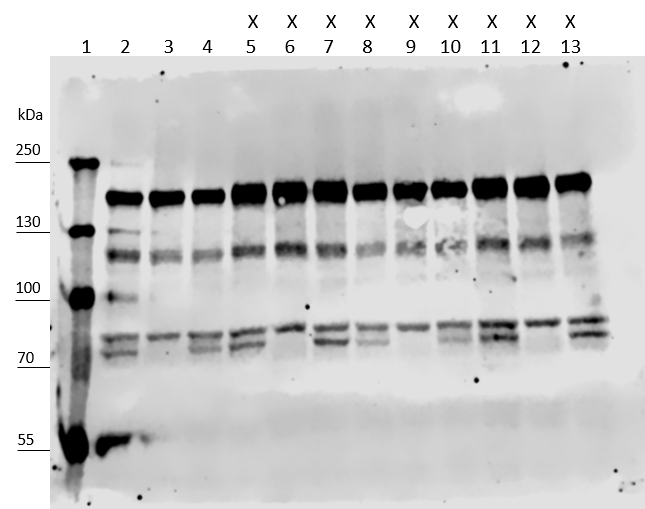


Fig. 3C Anti-V5 and anti-α-tubulin


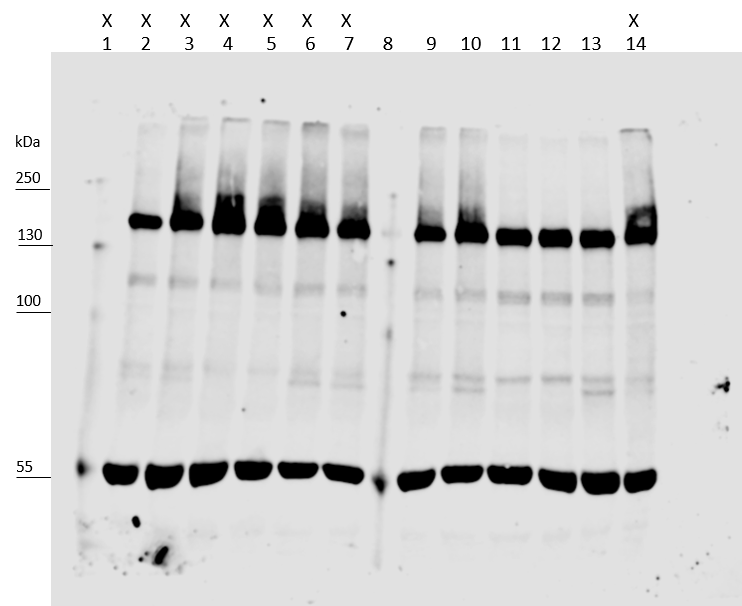


Fig. 3C Anti-V5 and anti-α-tubulin (higher exposure)


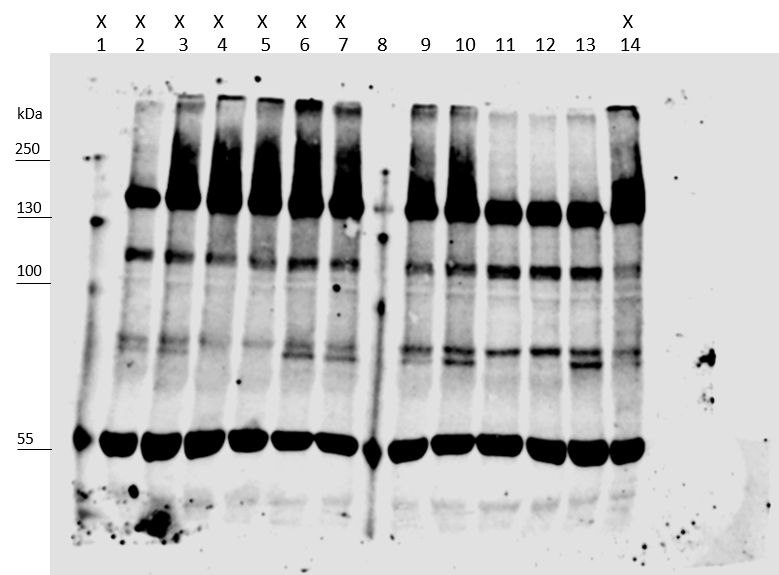


Fig. 3D Anti-RPTPα^Y789^


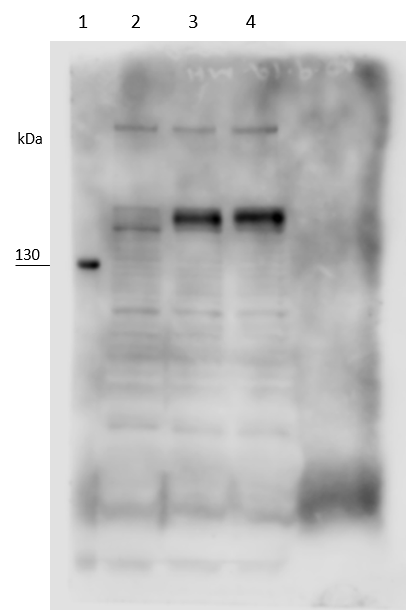


Fig. 3E Anti-pY Fig. 3E Anti-pY (higher exposure)


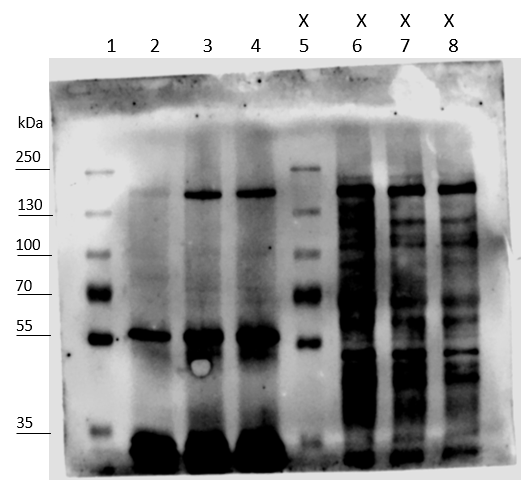

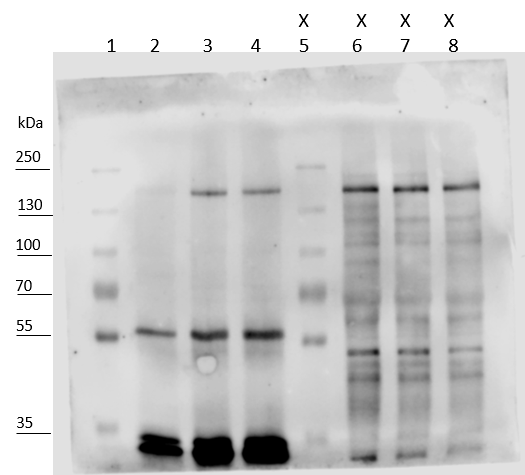


Fig. 3E Anti-V5


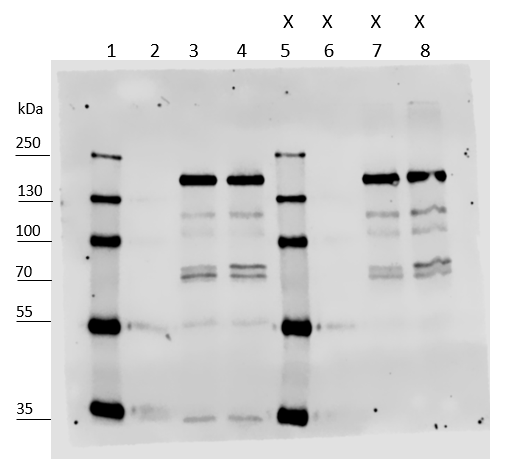


Fig. 4A Anti-V5


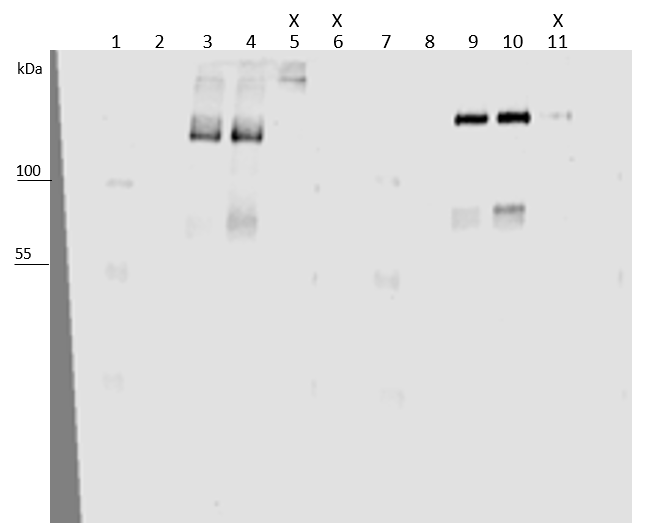


Fig. 4B Anti-FLAG

^
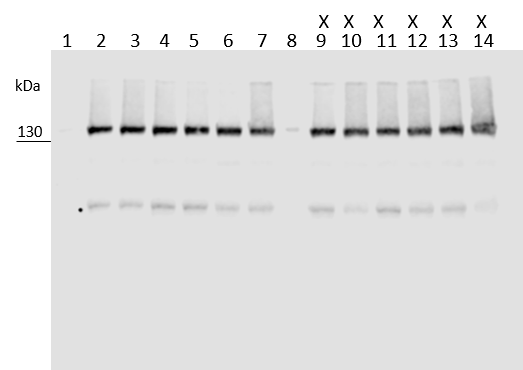
^

Fig. 4B Anti-npSrc^Y527^

^
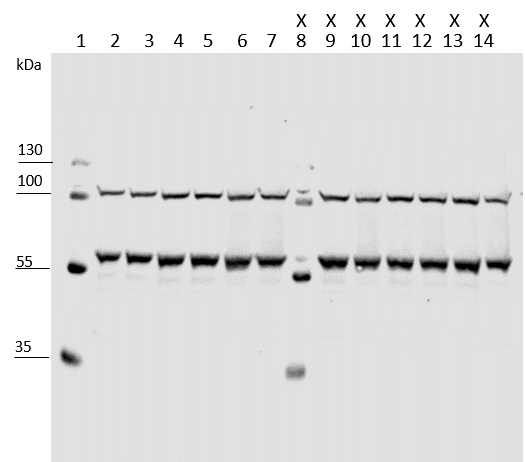
^

Fig. 4B Anti-tSrc


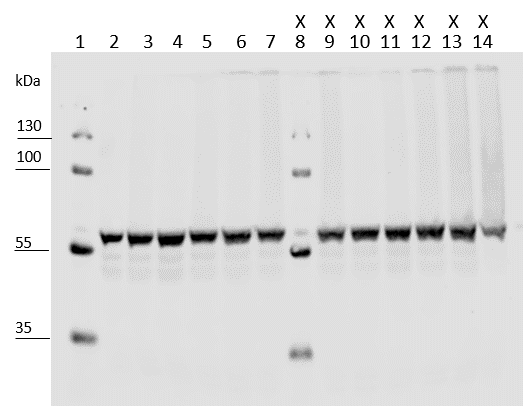


Fig. 4B Anti-pERK1/2


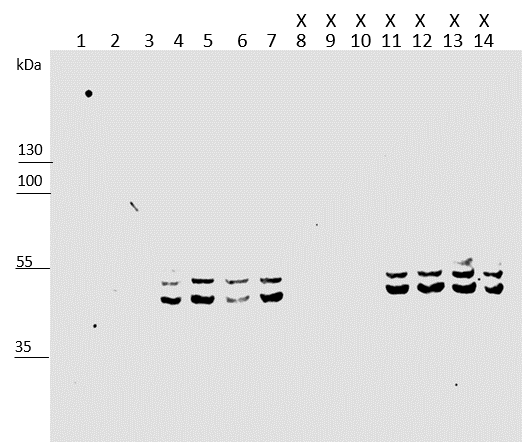


Fig. 4B Anti-α-tubulin


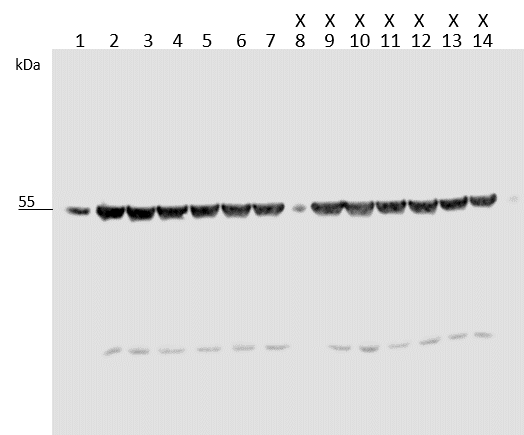


Fig. 5A Anti-pSrc^Y416^ replicates 1-2 Anti-tSrc C

1 2 3 4 5 6 7 8 9 10

1 2 3 4 5 6 7 8 9 10­


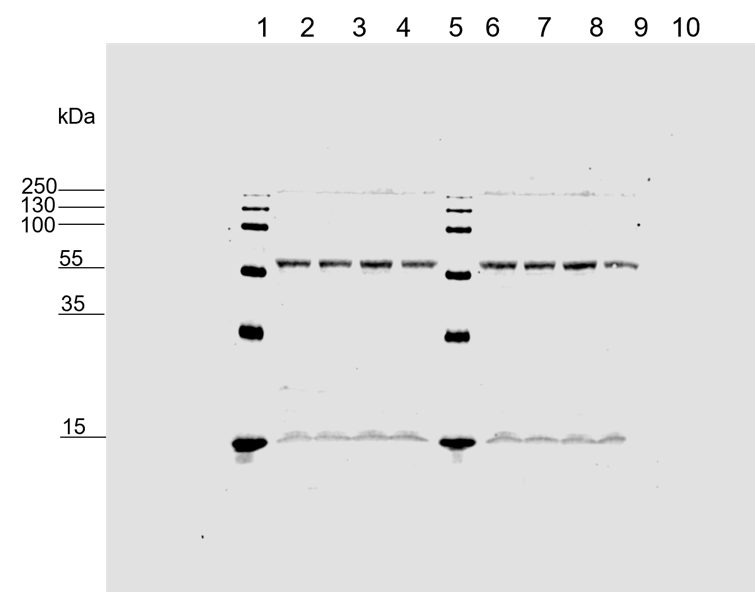

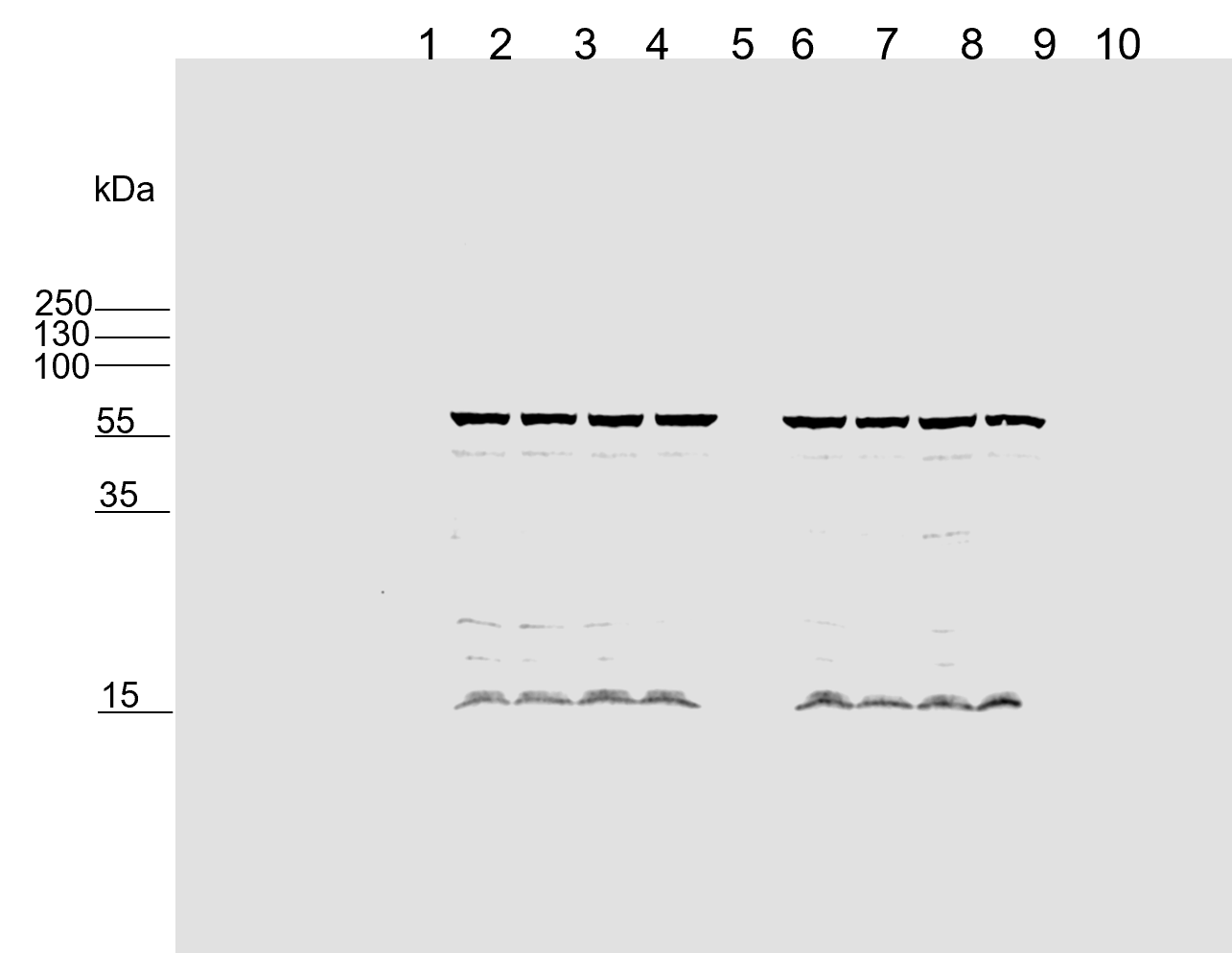


Anti-pSrc^Y416^ replicates 3-4 Anti-tSrc A

1 2 3 4 5 6 7 8 9 10

1 2 3 4 5 6 7 8 9 10


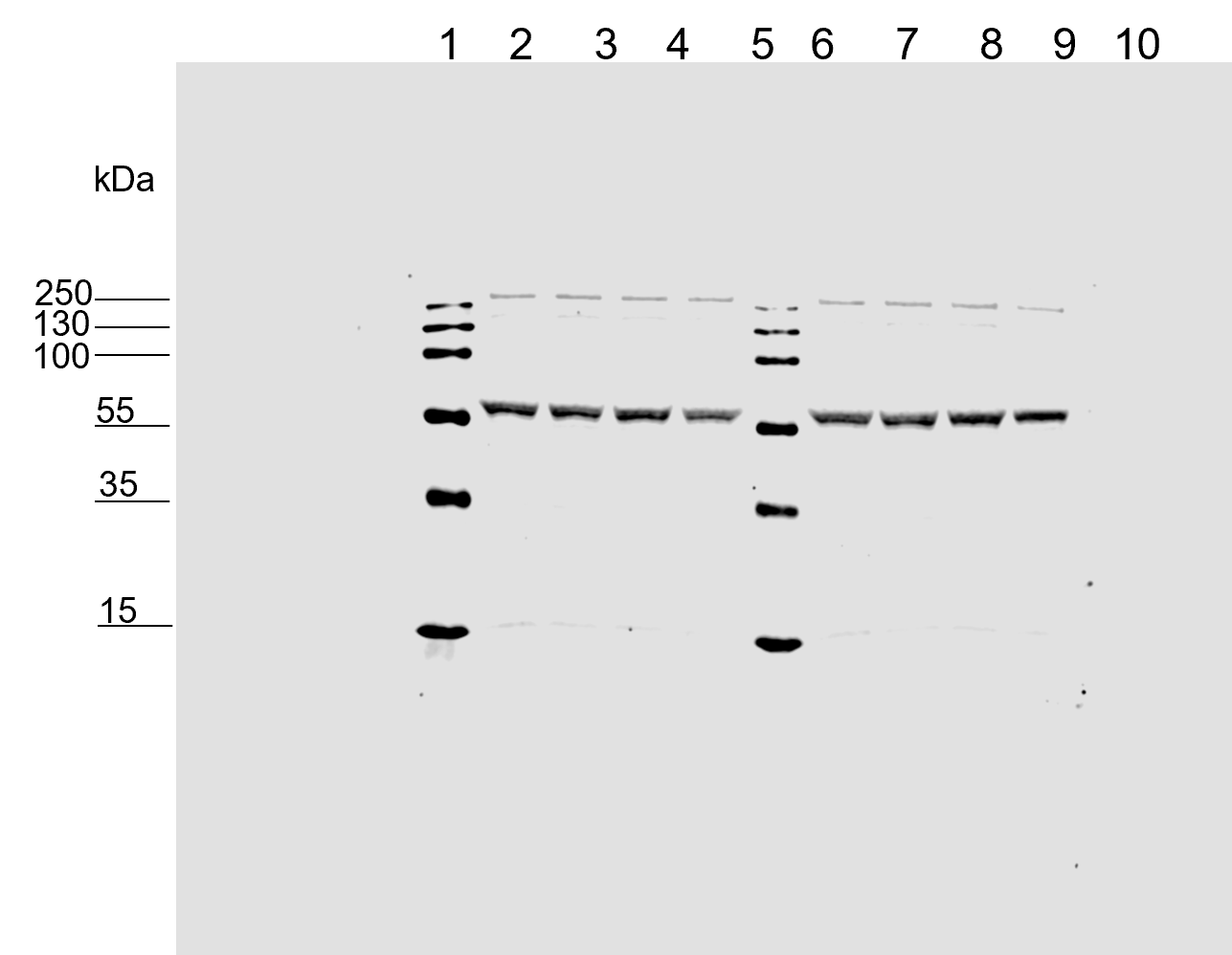

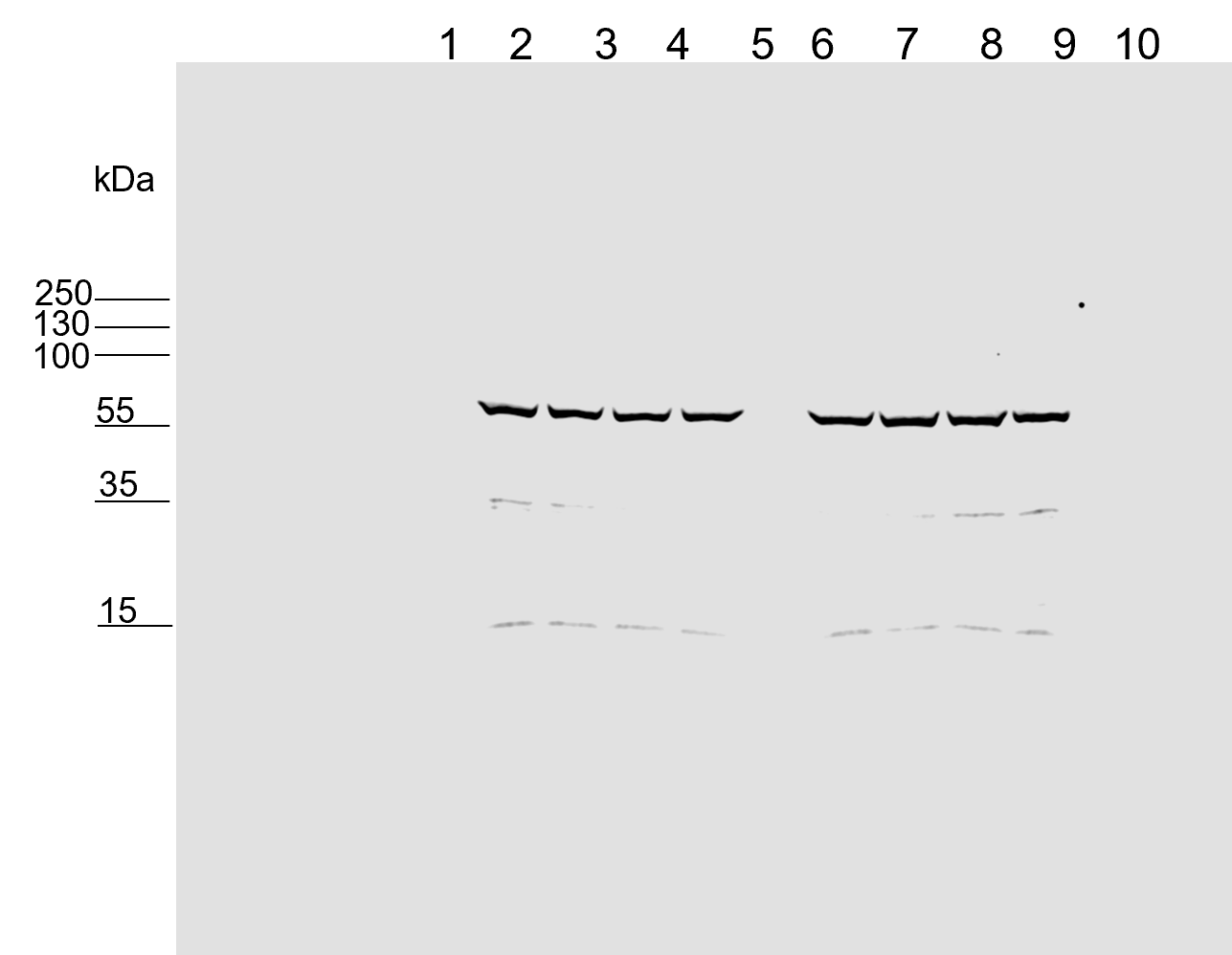


Anti-pSrc^Y416^ replicate 5 Anti-tSrc B

1 2 3 4 5

1 2 3 4 5


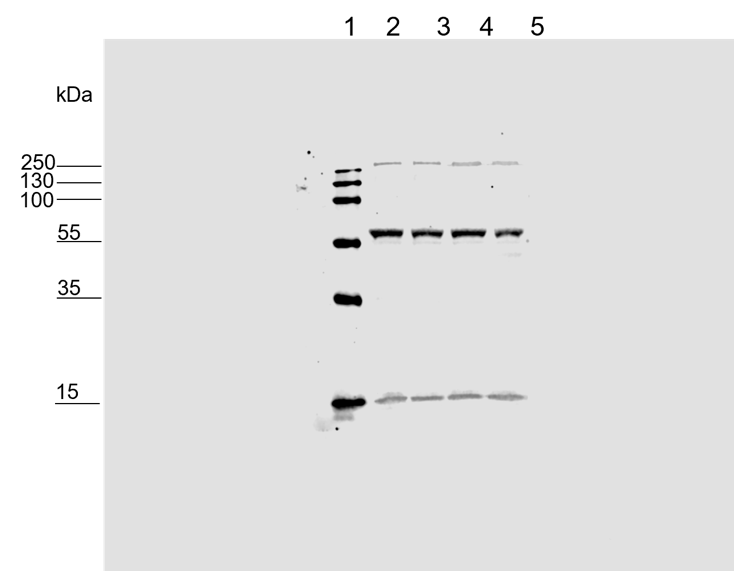

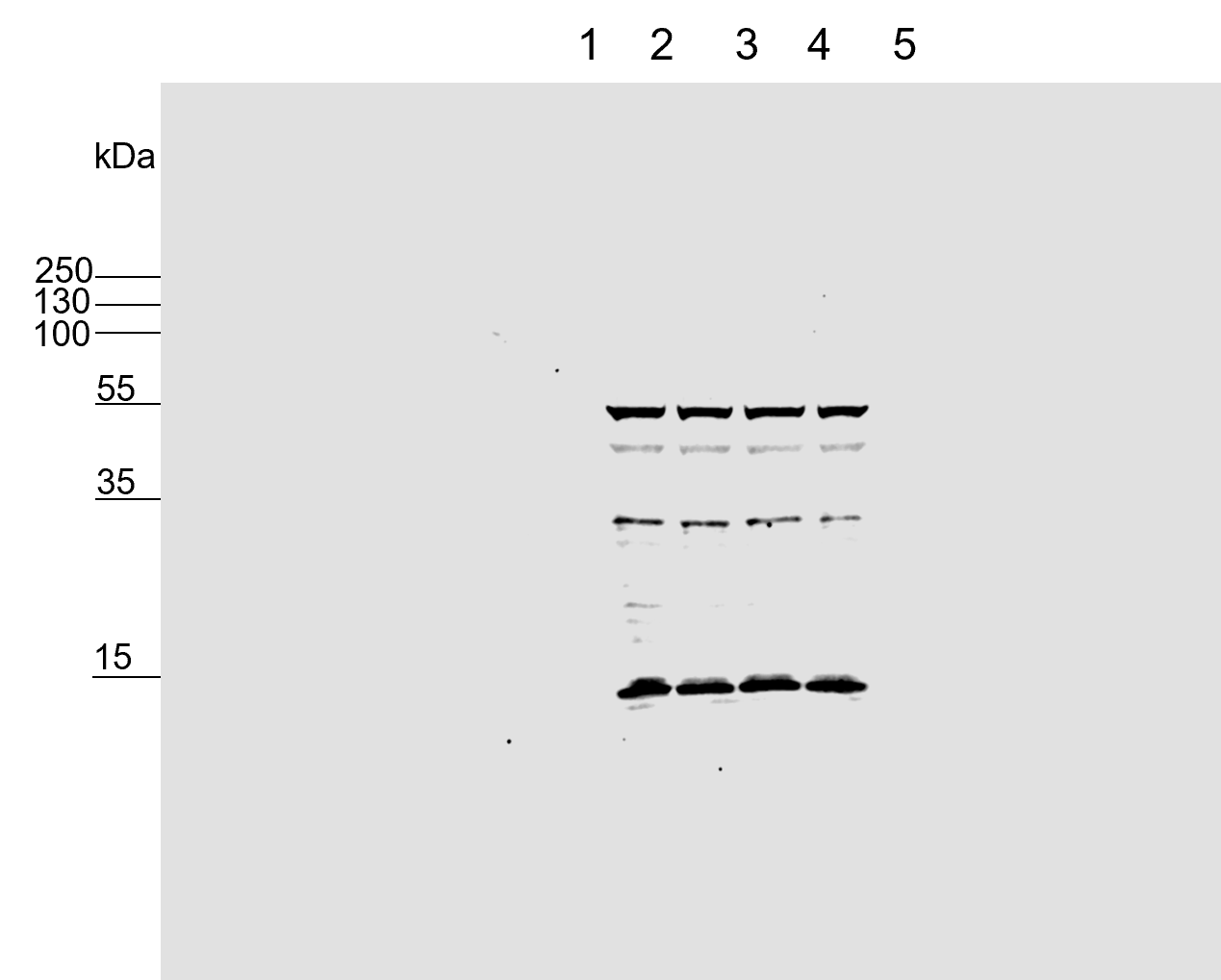

Supplement: S1 Raw images — (DOCX) [file pone.0271499.s002.docx]
