# Supplementary material for: Does a rare mutation in PTPRA contribute to the development of Parkinson’s disease in an Australian multi-incident family?
Source: PLoS One. 2022 Jul 28;17(7):e0271499. doi: 10.1371/journal.pone.0271499 (PMC9333306; doi:10.1371/journal.pone.0271499)
Supplement: Attachment — Submitted filename: PLOSone Comments to the Author_v3.docx [file pone.0271499.s003.docx]

Reviewer #1: 

*The study in principle is of interest. However, even as an initial study, it has several problems. First, how likely is a causal role of RPTPα for the development of Parkinson's disease when the new sequence data (page 12) show a similar prevalence of the mutant in patients and controls (~1:1000)?*

The reviewer raises a valid question and which we had considered carefully before composing the manuscript. While the International Parkinson’s Disease Genomics Consortium and United Kingdom Biobank data does suggest similar prevalence rates, we describe the limitations of this data. Referring specifically in the discussion, *“(1) only 10% of participants are over 80 years of age. (2) 3,602 participants had PD, however,19,460 parents of participants were reported to have PD (Bycroft et al., 2018; Van Hout et al., 2020). Carrier status of these participants was unknown. (3) The dataset had disclosed 30.3% of participants were related up to the third-degree (Bycroft et al., 2018), indicating frequency of rare variants may be exaggerated due to these familial cluster*s”, the age of participants from the UKB, unknown carrier status of participants with PD affected relatives and the high rate of third-degree or closer relationships decreases the reliability of this dataset as a true measure of the rate of the rare mutation in an unaffected control population. Nevertheless, we have included this data for transparency. Furthermore, we also propose additional factors, such as the other 2 rare mutations, other genetic or environmental factors, may operate in synergy with the aberrant RPTPα functionality to increase disposition of disease, which increases the complexity of the variant-disease relationship. Referring to the discussion text: which has been adjusted to now read “Further, this could be further exacerbated by other rare variants, such as the *ARL14EP* and *HYDIN* SNVs and/or other genetic background and environmental factors.“

We also describe in the discussion, to assess the risk of this variant in isolation, we would require a balanced case-control study of 120,000 participants, which unfortunately, is larger than currently available IPDGC or UKB datasets or feasible for us to perform.

*Next, the cDNA of RPTPα was newly cloned. How good was the sequence characterized? The transiently expressed protein shows an abnormal molecular weight. RPTPα is normally found as a protein of 130 kD and not 180.*

In our study we have used RPTPα SV1(793AA protein, transcript ID ENST00000216877.10, also termed PTPα123 (Kapp et al. 2012)) and not RPTPα SV4 (802AA protein, transcript ID [ENST00000399903.7](https://asia.ensembl.org/Homo_sapiens/Transcript/ProteinSummary?db=core;g=ENSG00000132670;r=20:2873420-3039076;t=ENST00000399903), also termed PTPα132 (Kapp et al. 2012)). We have fully sequenced the open reading frame and confirmed that it is correct. Furthermore, the observed MW of the mature full length RPTPα SV1 @~160kDa (between the 250 and 130 kDa bands) is consistent with (Yao et al. 2017) which studied RPTPα SV1 – as designated by the Y789F mutation (Y798 in SV4) Fig 6E and supplemental figure S5 A, B – PTPRA-FLAG migrating between 250/130 kDa and the “precursor” ~120 kDa which is equivalent to what we also observe in our study.

To make it absolutely clear which splice variant has been used the materials and methods now reads “RPTP⍺ (splice variant 1; transcript ID ENST00000216877.10) was amplified from HEK293T cDNA”

*The proteolytic processing of the extracellular domain of RPTPα occurs only for a splice variant containing an additional 9 amino acids in the extracellular domain.*

We initially believed the bands to be produced by RIP and after reanalysing the data have completely rewritten this section. Aside from our study, RPTPα SV1 cleavage bands also present @ 68 and 63 kDa Fig 1A (Kapp et al. 2012). In this paper the 63 kDa band is interoperated to be produced by calpain cleavage and the 68 kDa “has the size of the PTPαintra and might occur right at the plasma membrane” was not studied further.

In our hands the equivalent bands at ~75kDa (C1), which may be cleaved close/at the membrane is not stimulated by PMA or inhibited by TAPI2/BB94 indicating that it is not generated by metalloproteinase. However, the ~70kDa (C2) band, is stimulated by PMA and is completely inhibited by TAPI2/BB94 indicating that although it is generated by calpain cleavage it first requires cleavage by a metalloproteinase.

*Has this variant been cloned? If so, why then do we not see a total of three processed fragments, as has been published: 75 kD for the membrane anchored form, and 63 and 68 kD for the cytoplasmic products of calpain cleavage? Why is there only little stimulation of the metalloprotease by PMA (Fig. 3B)? And why was the metalloprotease not stimulated with POV which works better? Similarly, the calpain protease was not tested by stimulation with a Ca-ionophore. Since the authors claim the 70 kD protein fragment to be the product of the metalloprotease, this would have been essential.*

We decided to use PMA as it stimulated equivalent if not better cleavage of RPTPα SV4 (Kapp et al. 2012 Fig 1C and Fig 2A, termed TPA). Our hypothesis was that the R223W fragment was being generated by metalloproteinases and that PMA would increase its abundance. We have performed MG132 treatment and conclude that the 70kDa (C2) fragment is produced by calpain cleavage as its abundance is decreased by MG132 treatment (Figure 3B). However, PMA stimulates the generation of the 70 kDa (C2) band and this generation is completely blocked by TAPI-2, indicating an initial metalloproteinase step happens prior to calpain cleavage.

*Finally, the term "regulated intracellular proteolysis" (p10) is inappropriate for an extracellularly cleaving metalloprotease.*

We thank the reviewer for their comment and we agree and have adjusted the whole section accordingly using the terminology C1 and C2 for the two cleavage fragments.

*The immunofluorescence shown in Fig. 2C is of very low quality and does not allow to see a difference between overexpressing and background cells, making it difficult to accept that trafficking of the mutant protein is normal. The phalloidin staining is a complete failure.*

We have completed new imaging with just anti-FLAG, with higher quality images. A non-transfected cell control is added to demonstrate the specificity of the antibody staining.

*The authors find no tyrosine phosphorylation on the proteolytically processed fragments and conclude that these do not possess catalytic activity. However, they do not prove and not even give a reason for their hypothesis.*

We thank the reviewer for their comment and have addressed this with the following text and the results section now reads “Furthermore, when we investigated the phosphorylation status of the C1 and C2 fragments we observed that they were not phosphorylated (Fig 3C-D) indicating that these fragments are potentially catalytically inactive, as Y789 phosphorylation has been shown to be required for phosphatase activity against SFKs.”

And discussion section with the following text

“The absence of phosphorylation at Tyr789 in these cleaved fragments suggests they may be catalytically inactive against SFK substrates. The phosphorylation of Tyr789 provides the binding site for the SH2 domain of Src during Src activation. Indeed, mutation or dephosphorylation of this residue blocks RPTPα ability to dephosphorylate Y527 in Src both *in vitro* and *in vivo*.”

*In addition, the authors provided the same image in Fig. 3A and 3D. This becomes obvious when looking at the complete blot in the supplemental figure.*

We agree with reviewer #1 and Fig 3D was accidently used also for Figure 3A. We have corrected Figure 3A (and supplemental figure) with the correct image.

*In Fig. 4A, dimerization of the mutant phosphatase is tested. As a control, a delta-wedge mutant is added. This protein is hardly expressed which should at least be discussed.*

We have addressed this with the following text and the section now reads “The detection of RPTPα^Δwedge^ by immunoblot was consistently lower than the full-length proteins in our experiments (data not shown) but nonetheless demonstrated the importance of the wedge region but not R223 in RPTPα homodimerisation.”

*Finally, in Fig. 4B the effect of the mutant on Src activation is investigated. Phosphorylation of Src-Y527 is more strongly present 10 min after serum stimulation than in presence of the wild type form. This is a surprising result but it is also surprising that the inhibitory Src-Y527 phosphorylation increased at all after serum treatment for both phosphatase variants. The considerably varying levels of phosphatase expression may be one explanation, however, also the level of activating phosphorylation of Akt is higher before stimulation than after 5 and 10 min. This rather points to a general experimental problem. It would have been less error prone to establish cell lines overexpressing RPTPα than performing transient expression experiments. Last, the tubulin blot in Fig. 4B, right panel is part of the total blot shown for RPTPα in the supplemental figure. How can it appear as a separate blot without additional bands as the last figure of the supplemental data?*

To address this more specifically we generated stable HEK293 RPTPα WT and R223W cells, performed EGF stimulation, pERK1/2 analysis and have rewritten the entire section accordingly.

Reviewer #2:  Points:
*1. Src Tyr527 phosphorylation appears to be affected by expression of the R223W variant, in that serum stimulation results in a transient increase in Tyr527 phosphorylation for about 30 min in the presence of wild type RPTPalpha and only 10 min in the presence of the R223W variant (Fig.4). The authors claim that Src activity is affected differently by wild type and mutant RPTPalpha. This is not evident from the data provided. Tyr527 is an inhibitory phosphorylation site, which is phosphorylated by Csk. Activation of Src will lead to enhanced phosphorylation of the autophosphorylation site, Tyr416. Phosphorylation of Tyr416 is generally taken as a measure for Src activity. The authors should therefore reprobe their blots with Src pTyr416-specific antibodies to be able to conclude that Src activity is affected. It is noteworthy that both pTyr416 and pTyr527 are substrates of RPTPalpha.*

We have changed the experimental design to use stable expressing RPTPα cells, EGF stimulation and pERK1/2 activation.

*Minor points:
1. The last sentence of the main text does not read well: “This results in a subtle, yet significant delay in activity potentially altering SFK signalling would be altered in the brains of affected patients.”*

Due to the new data this sentence has been removed

*2. Ref 9 appears to be the same as ref 20.*

We thank the reviewer for their feedback, we have removed the duplicate reference accordingly.
