# Supplementary material for: Does a rare mutation in PTPRA contribute to the development of Parkinson’s disease in an Australian multi-incident family?
Source: PLoS One. 2022 Jul 28;17(7):e0271499. doi: 10.1371/journal.pone.0271499 (PMC9333306; doi:10.1371/journal.pone.0271499)
Supplement: Attachment — Submitted filename: PTPRA_plosone_response_v4.docx [file pone.0271499.s004.docx]

Reviewer #1: The revised version of the manuscript does provide only limited improvements for the understanding of the R223W mutation in PTPa.

*- A relevance for PD is purely speculative*.

We are not claiming PTPRA^R223W^ is a causative factor in the development of PD, merely that it was found in a patient with PD and the mutation has an effect on the protein. The SNV results in aberrant Src signalling which is addressed in the final paragraph “Nonetheless, this putative PD associated mutation, *PTPRA* p.R223W does generate a functional protein which is cleaved by an unknown protease to a greater extent than the wild-type protein and results in impaired Src activation. Whether this contributes to increased risk of PD alone, or in concert with the other SNVs, still remains to be determined.”

*- The aberrant molecular weight of PTPa has not been explained and still is not pointed out in the manuscript. Yao et al. (ref 25) also use a carboxyterminal tag of PTPa and in Fig. 4B do show a Western blot with a PTPa with regular molecular weight (about 130 kD). In contrast to the authors’ statement Yao et al. do not show a MW for the PTPa in Fig 6E but indeed do so in their Figure S5 and thus give two different MW of the phosphatase. Nevertheless, I suggest to point out the strong change of MW of the tagged PTP in the manuscript.*

We thank the reviewer for their thorough analysis of the literature. We agree that there is discrepancy in the literature for the observed MW of RPRPα and have added the following to the text.

“While the observed MW of RPTPα-V5/Flag is approximately 30 kDa larger than what is most commonly reported in the literature^21–24^ it is not uncommon for the protein to be reported larger than 130 kDa^25–29^ regardless of number or position of tags. ”

*- Phosphorylation and activity of PTPa fragments C1 and C2: the exposure shown in Fig. 3E is to short to allow detection of tyrosine phosphorylation (see bottom part of 3E and the relative amount of FL and C1/C2).*

To address this, we have included a higher exposure image to Fig 3E. There is no tyrosine phosphorylation in the region of the C1 and C2 bands.

*The authors state that “Y789 phosphorylation has been shown to be required for phosphatase activity against SFKs (ref 24,25)“. However, there is no activity assay for PTP fragments in ref 25. The paper of Zheng et al. (ref 24) has shown full activity for full length mutated Y789F-PTPa towards Raytide and MBP.*

The authors again thank the reviewer for their thorough knowledge of the literature. Relevance of the Y789 to Src activity appears to still be debated in the literature, therefore we have added the following to the discussion:

“According to the tyrosine displacement model presented by Zheng and collegues^38^, pY789 of RPTPα is required to displace the Src SH2 domain, allowing access to pY527. However, several studies dispute this model, showing that Y789 phosphorylation is not necessary for pY527 dephosphorylation^29,41–43^. Meanwhile it has reported that activity of the Y789F mutant depended on substrate, with a pool of mutants still active against EGFR/Src, and PTPRA-potentiated ERK activation unaffected by Y789F mutants^32^. As no consensus has yet been reached as to the necessity of pY789, further work is required to verify the activity of C1 and C2.”

*Since the changed proteolytical processing of PTPa-R223W that yields C1/C2 fragments is now the major novelty in this manuscript, it would have been appropriate to investigate the processing and the activity of the PTPa fragments.*

The authors agree that investigation of the activity of the fragments would be appropriate, unfortunately it is outside the scope of this project. In addition, we now present new data showing hampered Src activation in the RPTPα R223W and R232W, and therefore a second point of interest for this SNV.

*- The following point is important but had been overlooked in the original review: The sentence in the manuscript “Interestingly, we discovered that the wedge region is indeed responsible for the stability of homodimers, as RPTPαΔwedge expressing cells were present almost completely as dimers“ is contradicting itself. If the wedge is important for dimerization, its deletion should abolish dimerization. This is in agreement with the finding of Jiang et al. (2000) that dimerization of PTPa was not possible when a similar deletion of the wedge region was made.
In addition to the low expression of the delta-wedge PTPa, the enhanced dimerization also needs to be discussed.*

We agree that our findings were in contradiction to the literature and we did not expect the results we obtained. However, we added the data because we thought it would help illustrate the assay. As the RPTPαΔwedge experiments do not add anything beneficial to the paper we have removed them from the manuscript and the figures.

- Data shown in Fig. 4B: here, newly generated cell lines have been employed to get more physiological data. These data show clearly that there is no change of activity towards Src-pY527 in the PTPa mutant. The authors have a similar point of view, as they state at the end of the Results section “These data suggest that the phosphatase activity of the R233W mutant is not hampered in comparison to RPTPαWT after EGF stimulation.” It is therefore surprising to find as a head line of the last chapter of the Results section “The RPTPαR223W mutation decreases activity against SFKs but doesn’t alter homodimerisation“. Or this sentence in the Discussion “Nonetheless, the PTPRAR223W variant had altered the normal protein function.”

The authors agree that consistency in our conclusions regarding protein function were not consistent in the manuscript and have now been corrected.

*Taken together, the revised version leaves the reviewer with the conclusion that the PTPa-R223W mutant has no meaning for PD, and the only cell biological change for PTPa is a slightly altered proteolytical processing of the phosphatase for which the mechanism and the meaning are unknown.*

With the new R223W and R232W data we think that this is addressed.

Reviewer #2: In the original manuscript, the authors concluded that the Parkinson’s disease-associated mutation they identified in PTPRA in an Australian family altered proteolytic processing and activity of RPTPalpha. Using stable lines in HEK293 cells, they now report that SRC Tyr527 phosphorylation and ERK/MAPK phosphorylation were not affected. Proteolytic cleavage of the mutant was affected, but the functional consequences are not evident. Combined with incomplete penetrance of the mutation, the conclusion that this mutation represents a SNP that does not have functional implications would be equally valid.

*SRC family kinases are the most prominent substrates of RPTPalpha. As indicated in my original review, the authors should assess Src Tyr416 phosphorylation as a read-out for SRC activity and hence for signaling downstream of RPTPalpha. If Tyr416 phosphorylation is not altered in response to the mutation, there is little or no news value in this report.*

The authors thank the reviewer for their insightful commentary. We had previously encountered issues with successful resolution of pSrcY416 by western blot. Similar problems were resolved in a separate project with a change in the lysis procedure. We have now performed pSrc^Y416^ immunoblots on RPTPα^R223W^ and discovered attenuated Src activity. We have also generated RPTPa-Sv4^R232W^ (the longer isoform) stable cells which also have attenuated Src activity. These new data suggest that the patient SNV does indeed impact RPTPα and Src signalling.
