# Supplementary material for: Does a rare mutation in PTPRA contribute to the development of Parkinson’s disease in an Australian multi-incident family?
Source: PLoS One. 2022 Jul 28;17(7):e0271499. doi: 10.1371/journal.pone.0271499 (PMC9333306; doi:10.1371/journal.pone.0271499)
Supplement: Attachment — Submitted filename: PTPRA_plosone_response_v5.docx [file pone.0271499.s005.docx]

**Response to Reviewers**

Reviewer #1: The current revision does not provide relevant new insights or improve the manuscript essentially.

*A misleading statement is found in the rebuttal when the authors state “We are not claiming PTPRAR223W is a causative factor in the development of PD”. Why then do they mention PD 13x in Abstract and Introduction alone, if not for nudging the reader to see a role for PTPalpha in the development of PD?*

We agree that the language in our response could have been clearer. We are not claiming that PTPRA^R223W^ is the sole causative factor contributing to PD in this family. It likely increases risk of development, particularly in concert with the other identified mutations or environmental factors. We are not claiming that possessing the R223W mutation is a one-way ticket to PD. This is common in PD genetics for example even though SNCA and LRRK2 are robustly linked to PD, they are not 100% penetrant but are strong risk factors for developing PD (Delcambre et al., 2020; Trinh, Guella, & Farrer, 2014). This is covered by the following sentences in the discussion “While parkinsonism status of participants of gnomAD is not disclosed, it would be mistaken to assume that all *PTPRA* p.R223W carriers develop PD. Rather, we hypothesise that in this family, additive to their other genetic and environmental risk factors, the perturbed activity of the protein due to this variant conferred risk for developing PD before the age of 80 years.” and the last 2 sentences of the discussion “Nonetheless, this putative PD associated mutation, *PTPRA* p.R223W does generate a functional protein which is cleaved by an unknown protease to a greater extent than the wild-type protein and results in impaired Src activation. Whether this contributes to increased risk of PD alone, or in concert with the other SNVs, still remains to be determined.”

*- Fig. 3E: it is surprising that in 3E the longer exposure generates considerable general darkening of the blot whereas the blot in 3C gets lighter ?*

Figure 3C has clear and low intensity bands in the region of interest. Only moderate over exposure was required to visualise them better and the brightness/contrast was adjusted in ImageStudio after acquisition of the longer exposure. 3E has no noticeable bands and we have supplied the most over exposed image we have to illustrate this.

*- Fig. 5: The analysis of the densitometric scanning is statistically not significant. Therefore, the statements about “percentages of” are meaningless. When looking at Fig. 5B and seeing the deviation of the mean, it just brings out a laughter when the authors state in the Results section values of 80.6±0.09% and 54.3±0.21%.
As Fig. 5 has been included to overcome the deficit in analysis of PTP cleavage (statement of the authors in the rebuttal) this also flops.*

As the reviewer has had many issues with this data we have completely redone the experiments and to eliminate all sources of ambiguity we completed these analyses on single blots, using both rabbit anti-pSrc416 and mouse-anti-tSrc. Densitometric analysis revealed a significant reduction in pSrc416 phosphorylation in cells expressing mutant splice variant four RPTPα. A reduction in pSrc416 phosphorylation was apparent but not significant for the splice variant 1 RPTPα in the current experimental paradigm of 5 independent replicates. We have supplied all the blots from 5 independent replicates for complete transparency of these results (S1_raw_images.pdf).

Delcambre, S., Ghelfi, J., Ouzren, N., Grandmougin, L., Delbrouck, C., Seibler, P., … Grünewald, A. (2020). Mitochondrial Mechanisms of LRRK2 G2019S Penetrance. *Frontiers in Neurology*, *11*, 881. https://doi.org/10.3389/fneur.2020.00881

Trinh, J., Guella, I., & Farrer, M. J. (2014). Disease penetrance of late-onset parkinsonism: A meta-analysis. *JAMA Neurology*. https://doi.org/10.1001/jamaneurol.2014.1909
